# Endogenous Galectin-1 in T Lymphocytes Regulates Anti-prostate Cancer Immunity

**DOI:** 10.3389/fimmu.2018.02190

**Published:** 2018-09-26

**Authors:** Enrique Corapi, Gustavo Carrizo, Daniel Compagno, Diego Laderach

**Affiliations:** ^1^Laboratorio de Glico-Oncología Molecular y Funcional, Departamento de Química Biológica, Facultad de Ciencias Exactas y Naturales, Universidad de Buenos Aires, Buenos Aires, Argentina; ^2^CONICET–Instituto de Química Biológica de la Facultad de Ciencias Exactas y Naturales, Buenos Aires, Argentina; ^3^Departamento de Ciencias Básicas, Universidad Nacional de Luján, Buenos Aires, Argentina

**Keywords:** prostate cancer, endogenous galectin-1 in lymphocytes, cancer immunotherapy, tumor microenvironment, tumor immune escape

## Abstract

The identification of effective new therapies for prostate cancer (PCa) requires a better understanding of the multiple molecular interactions between tumor cells and their associated microenvironment. In this context, galectin-1 (Gal-1) is a key molecule in the determination of the prostatic carcinoma microenviroment; therefore, it is essential to understand all the molecular processes in which this protein is involved. Most of the previous studies found in the literature have focused on the microenvironment remodeling properties of tumor-secreted Gal-1, through its interactions with the glyco-receptors at the cell membrane and the extracellular matrix. This report shows original aspects of the lectin by focusing on the role of lymphocyte endogenous Gal-1 in controlling anti-prostate tumor immunity. Using a murine preclinical model of prostate cancer, our results demonstrate that endogenous Gal-1 in lymphocytes modulates their proliferative rate and cytotoxic function in conditions of high extracellular Gal-1 concentration, mainly derived from tumor cells. In such conditions, the absence of Gal-1 in T lymphocytes potentiates anti-tumor immune responses. Further studies demonstrated that endogenous Gal-1 in CD4+, but mainly in CD8+T cells, acts as a negative regulator of anti-tumor immunity. In conclusion, prostate tumors require Gal-1 in lymphocytes to evade immune responses. This report lays the foundation for an original immunotherapy strategy for prostate cancer.

## Introduction

Prostate cancer (PCa) is a major challenge for public health worldwide; epidemiological studies place it as the second-screening and the fifth-leading cause of cancer death in adult men (1). Suitable treatments currently exist for this type of cancer if detected early; in contrast, new treatment options are required for advanced stages of the disease (2). Immunotherapy is a validated alternative for the advanced stages of PCa, e.g., Sipuleucel-T (Provenge®) is the first immune-based therapy approved by the US Food and Drug Administration for this type of cancer (3). In addition, several immunotherapy strategies are currently being evaluated for this and other types of cancers involving active immunization against tumor-associated antigens and blocking immune checkpoint molecules (4, 5). In this context, Galectin-1 (Gal-1) is a clue molecule secreted by different types of cancers to promote a tolerogenic microenvironment (6). These tolerogenic effects occur locally at the primary tumor site but also at a distance since Gal-1 is found soluble in plasma and is transported inside microvesicles, such as exosomas (7). Indeed, most of the immunological studies about Gal-1 are focused on its lectin properties resulting from its interaction with β-galactosides and promotion of lattice formation with membrane receptors and extracellular matrix proteins [reviewed in (8)]. This kind of interactions induces lymphocyte apoptosis after activation (9–11), T cell exhaustion (7) and inhibits T-cell migration across endothelial cells (12); actions that favor the tumor-inefficient immune responses.

However, Gal-1 is also expressed intracellularly in several cell types; it has been reported on transformed tumor cells (13–16), regulatory T cells (17, 18) and antigen-presenting cells (19, 20), where the endogenous Gal-1 plays a role as a regulator of the anti-tumor immune responses. Effector T lymphocytes also express endogenous Gal-1; the absence of Gal-1 in lymphocytes promotes a Th1 response during parasitic diseases (21, 22). Few data are available about the role of the endogenous Gal-1 in the control of cytotoxic CD8+ T cell responses. On one hand, Gal-1 negatively regulates CD8+ T cell expansion, survival upon activation, and function fate (23). On the other hand, another study has demonstrated that Gal-1-deficient mice have impaired *in vivo* anti-viral CD8+ T cell–mediated immune responses (24). There are no data about the role of endogenous Gal-1 expressed by CD8+ T lymphocytes in the control of the anti-tumor properties. However, translating the second concept, in which the absence of Gal-1 impairs immune cytotoxicity, seems controversial, due to the compelling evidence in the literature. In fact, Lgals1-/- mice have Foxp3+ Treg cell suppressive dysfunction (17) and are prone to autoimmunity (10, 19, 25–27). More interestingly, the injection of tumorigenic cell lines into Lgals1-/- mice demonstrated reduction of tumorigenesis (28, 29), proving the relative importance of the stroma (including immune cells) in determining tumorigenesis potential. Given this scenario, this research aimed to challenge the role of Gal-1 as an endogenous modulator of T cell properties in PCa. Although expressed at low levels in lymphocytes, endogenous Gal-1 plays a major role in the control of the lymphocyte anti-tumor functions in a PCa context. The results allow us to propose the modulation of the endogenous load of Gal-1 in T cells as a novel immunotherapeutic strategy for PCa.

## Materials and methods

### Animals

Animal procedures complied with the Guidelines for the Welfare of Animals in Experimental Neoplasia (UK) and approved by the University of Buenos Aires's Institutional Animal Care and Use Committee (IACUC; FCEN protocol #2014-038). Six to eight-week-old male C57BL/6 mice were housed in the animal facility of the School of Sciences, University of Buenos Aires (Argentina). Lgals1-/- mice (originally produced by Dr. F. Poirier, France), were provided by Dr. Gabriel A. Rabinovich (Argentina). Ly5.1 C57BL/6 (CD45.1) mice were provided by Dr. Sophie Ezine (CNRS CDTA; Orleans, France). Athymic nude *Foxn1*^*nu*^ mice were acquired from the animal facility of the Veterinary School, National University of La Plata (Argentina).

### Cell culture

Murine PCa cell line TRAMP-C1 (obtained from ATCC) was cultured in Dulbecco's Modified Eagle Medium (DMEM; Invitrogen), 10% heat-inactivated FBS (Gibco), antibiotics (1 U/ml penicillin, 1 μg/ml streptomycin, 2.5 ng/ml amphotericin) and 0.25 U/ml Insulin (Humulin N; Eli Lilly and Co). Cell morphology was routinely evaluated, and cells were periodically examined for androgen sensitivity (MTT assay) and mycoplasma contamination (PCR). Lymph node cell primary cultures were carried out in proliferation medium: RPMI1640 (Invitrogen) containing 10% heat-inactivated FBS (PAA), 1 unit/mL penicillin, 1 μg/mL streptomycin, 2.5 ng/mL amphotericin B, 2 mM L-glutamine and 2 × 10^−5^ M β-mercaptoethanol.

Stable Gal-1 downregulated TRAMP-C1 cell line was produced by transduction with a shRNA lentivirus previously reported (30). Briefly, after 1 week, transduced (GFP+) cells were purified by cell sorting using a FACSAria II cytometer (BD Bioscience). Purification of the transduced cells was carried out if GFP^+^ cells did not exceed 20%, in order to minimize the number of viral integrations and thus guarantee a minimum perturbation of the genome. As previously reported, this shRNA sequence produced 85% of Gal-1 down-regulation at the protein level.

### Murine samples

Lymph node (brachial, axillary, inguinal and mesenteric; BAIM) samples were harvested and single-cell suspensions were obtained by mechanical disruption.

### Cell purification and immunoblotting

CD3+T lymphocytes were purified (>98% purity) by cell sorting (FACSAria; BD Biosciences) using PE labeled anti-CD3 mAb (1452C11, BD Pharmingen). Antigen presenting cells (APC) were purified by adherence to plastic (>90% CD14+ purity). TRAMP-C1 cells were obtained from an exponentially growing culture. Cells were lysed in radioimmunoprecipitation assay (RIPA) buffer [50 mmol/L Tris–HCl pH 8, 150 mmol/L NaCl, 1% IGEPAL, 0.5% sodium deoxycholate, 0.1% SDS, 10 mmol/L EDTA, 1 mmol/L sodium vanadate, and Protease Inhibitor Cocktail Set III (Calbiochem)]. Equal amounts of protein (20 μg) were resolved by 15% SDS- PAGE, blotted onto polyvinylidene difluoride (PVDF) membranes (GE Healthcare), blocked with 5% bovine serum albumin (BSA, Sigma-Aldrich), and probed with anti-galectin-1 (H-45 1:500, Santa Cruz) or anti-β-actin (H196 1:1,000, Santa Cruz) antibodies. Specificity of the used antibodies had been previously evaluated by immunoblotting (30, 31). Bound antibodies were detected with peroxidase-labeled anti-rabbit total immunoglobulins (1:3,000; Sigma-Aldrich). Peroxidase activity was detected using a luminol-based method and chemiluminescence was determined using a Fuji Photo Film Analyzer. Images were analyzed using ImageJ software (NIH, USA).

### Lymphocyte proliferation, apoptosis and degranulation assays

For T-cell proliferation assays, 5 × 10^5^ CFSE-stained murine lymph node cells (2.5 μM, 5 min; Sigma-Aldrich) were seeded in a 96-well U-bottomed plate in proliferation medium. To obtain optimal co-stimulation, 10% APC (adherent splenocytes, 5 × 10^4^) was added to each well. 1 × 10^4^ mitomycin-arrested TRAMP-C1 cells (3-h treatment, 10 μg/ml; Sigma-Aldrich) were added into cultures to mimic a tumor microenvironment. For T-cell proliferation, cells were stimulated for 72 h with coated anti-CD3 antibody (1 μg/ml; 145-2C11 purified mAb) and proliferation assessed by CFSE dilution. In the case of experiences with purified cells, CD4+ and CD8+ T cells were purified to homogeneity (>95% purity) by cell sorting (FACSAria, BD Biosciences) using fluorescent-conjugated mAbs (anti-CD8, clone 53-6.7, anti-CD4 clone GK1.5; anti-CD3 clone 1452C11; all from BD Pharmingen), mixed at a 2:3 CD8:CD4 ratio and cultured in presence of APC, tumor and anti-CD3 stimuli as before. For transwell assays, 250,000 lymph node cells from Lgals1-/- and WT mice were co-cultivated separated by a 0.4 μm pore size transwell system (Falcon). The low chamber was loaded with 250,000 KO lymphocytes, 2,500 TC-1 and 25,000 spleen adherent cells (APC) whereas the top chamber of the transwell was loaded with 250,000 WT (CD45.1); 2,500 TC-1and 25,000 APC cells. Lymphocytes were activated by 0.25 μg/ml anti-CD3 mAb (145-2C11, used soluble to activate T cells in both chambers). After 72 h of co-culture the membrane were removed and the lymphocyte proliferation were assess by CFSE dilution in the CD8+ CD45.2+ gate. In all cases, average stage of division (AOD) was calculated as before (23). Briefly, percentage of cells in each CFSE division was calculated on live CD8+ T cells and inserted into the following equation:

AOD = {[(% undivided) + (2 × % division 1) + (3 × % division 2) + xxx)]/100} – 1.

Apoptosis was determined by Annexin V (BD Pharmingen) staining, performed as before (32). For T-cell degranulation, cells were stimulated for 48 h with coated anti-CD3 antibody (1 μg/ml; 145-2C11) and anti-CD107a was added (1 h, 37°C; 1D4B, BD Pharmingen) followed by an additional 4 h-culture with monensin (3 μM, Sigma-Aldrich). In all the cases, cells were then stained for CD8 (53-6.7, BD Pharmingen) in staining buffer (PBS 1% FBS, 0.01% sodium azide) for 30 min on ice. Flow cytometric analysis was performed in a FACSAria (BD Biosciences) and analyzed using the FlowJo software.

### Generation of experienced anti-tumor lymphocytes

Dendritic cells (DC) were prepared from C57BL/6 bone marrows as previously described (33). Briefly, bone marrow cells were flushed with ice-cold PBS and erythrocytes lysed using ACK buffer (8.7 g/L NH_4_Cl, 1g/L KHCO_3_, 0.05 mM EDTA, pH 7.3). Cells were cultured in DMEM 20% heat-inactivated FBS (Gibco), 1mM sodium piruvate, 2 mM glutamine and antibiotics (1 U/ml penicillin, 1 μg/ml streptomycin) (Invitrogen), in presence of 20 ng/mL mrGM-CSF and 10 ng/mL mrIL-4 (ImmunoTools). Cells were cultured for 5 days, with addition of GM+IL-4 every 2 days. Immature DCs were pulsed during 5 h with TRAMP-C1 lysates (4 freeze/thawing steps), and then matured with 2 ng/mL CpG-1826 (phosphorothioate 5'-TCCATGA*CG*TTCCTGA*CG*TT-3', IDT) and 20 μg/mL Poly-U (Sigma-Aldrich) during an overnight culture. To obtain experienced anti-tumor lymphocytes, wild type or Lgals1-/- mice were *s.c*. immunized with tumor lysate-pulsed dendritic cells. At day 7 pot-immunization, lymphocytes obtained from axillary, brachial and inguinal lymph nodes were used for immune reconstitution of nude mice.

### Tumor model in nude mice reconstituted with wt or lgals1-/- experienced lymphocytes

Six to eight-week-old male nude mice were *i.v*. reconstituted with immune cells from one experienced wild type or Lgals1-/- mice. The day after reconstitution, 1 × 10^6^ TRAMP-C1 cells (log phase of growth) were *s.c*. injected in mice in Matrigel (4–5 mg/ml; Corning). No changes in mice weights were detected during the protocol. Euthanasia was practiced when tumor volume reached 2500 mm^3^. Tumor size was measured every 2 days with caliper and calculated as *W*^2^ × *L*/2, where *W* = width and *L* = length.

### Statistical analysis

At least three biological replicates (i.e., independent experiments) were carried out in each case, unless stated otherwise. Data represent mean ± SD. Prism software (GraphPad) was used. Two groups were compared with the Student *t*-test for unpaired data (two-sided tests). *P* < 0.05 was considered statistically significant.

## Results

### Endogenous Gal-1 in lymphocytes negatively regulates their functional properties in a tumor context

**Figure 1:**
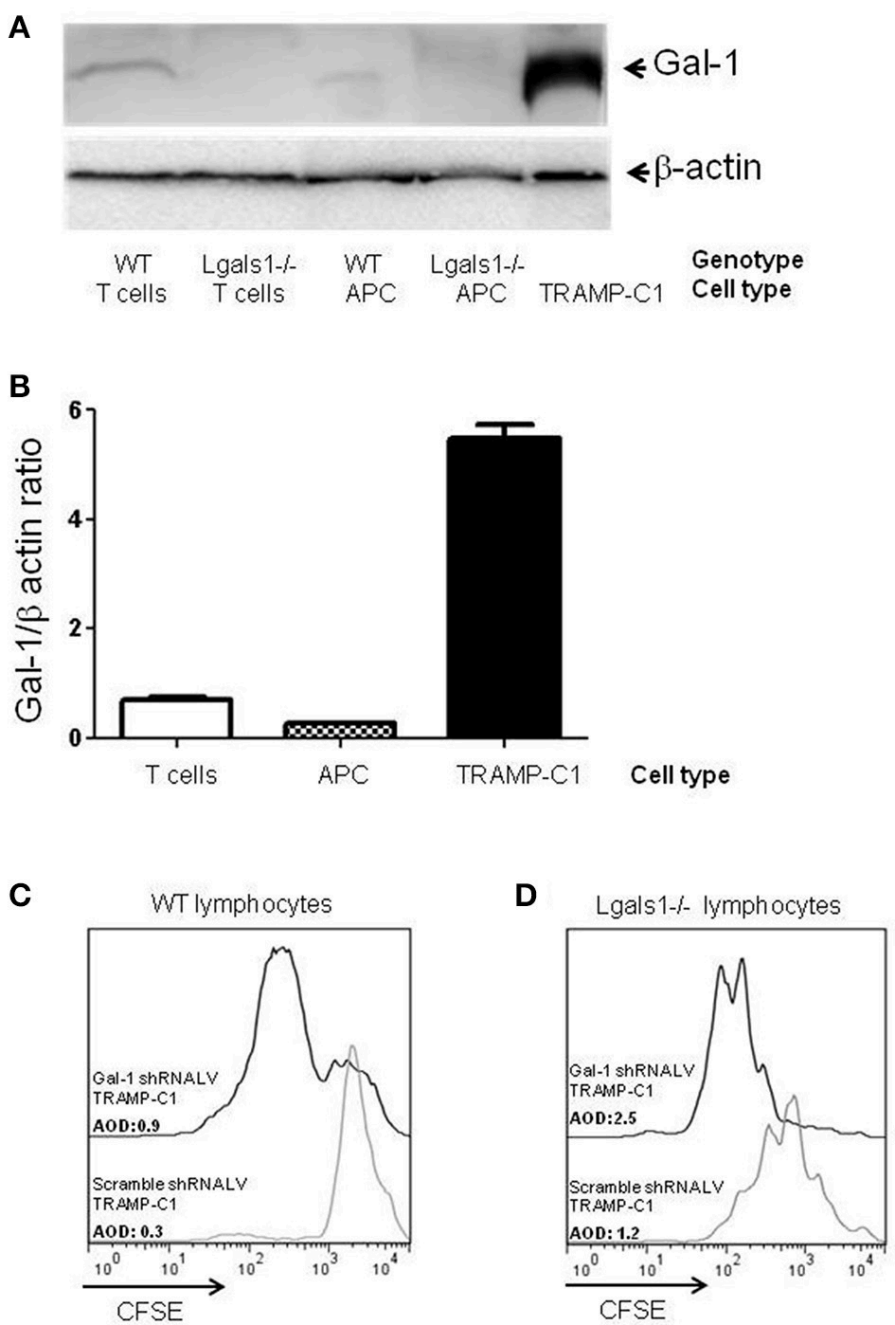
Comparison of Galectin-1 expression in immune cells. Gal-1 is detected by western blot in purified immune subpopulations. T lymphocytes were purified from lymph nodes, antigen-presenting cells from spleens. The specificity of the technique was verified using Lgals1-/- cells. Additionally, Gal-1 levels in TRAMP-C1 cells are comparatively shown. 20 μg of protein was resolved on 15% SDS-PAGE and blotted with commercial anti-Gal-1 antibodies. **(A)** Representative immunoblot (*n* = 3). **(B)** Mean Gal-1 levels in the three independent experiments. The results are expressed as Gal-1/β-actin ratio. **(C,D)** Immunosuppressive effect of endogenous Gal-1 expression in TRAMP-C1 tumor cells and in lymphocytes. Lymphocytes harvested from wild type **(C)** or Lgals1-/- **(D)** mice were polyclonaly activated in presence of 10% wild type APC and 1% of control or modified TRAMP-C1 tumor cells. Proliferation was measured at 72 h by CFSE dilution. Black line: TRAMP-C1 cells with downregulated Gal-1 (stable transduced, 90% of Gal-1 down-regulation-data not shown-). Gray line: TRAMP-C1 cells transduced with a control lentivirus. Representative of two independent experiences.

A vast literature in different animal models and experimental design are found describing the tumor cells as the main producer of Gal-1 (13, 15, 34–44). Regarding PCa, we previously described that Gal-1 is expressed at high levels in several human PCa cell lines and, more importantly, in patient prostatectomies, being upregulated during disease progression (30, 45). Gal-1 is also highly expressed in murine TRAMP-C1 cells (Figures 1A,B) and the downregulation of Gal-1 in these cells by RNAi reduces their immunosuppressive potential. In fact, the *in-vitro* polyclonal activation of lymphocytes in the presence of Gal-1 downregulated TRAMP-C1 led to higher proliferation of CD8+T cells (Figure 1C). Consequently, Gal-1 is an important immune escape mechanism activated by the tumors.

**Figure 2:**
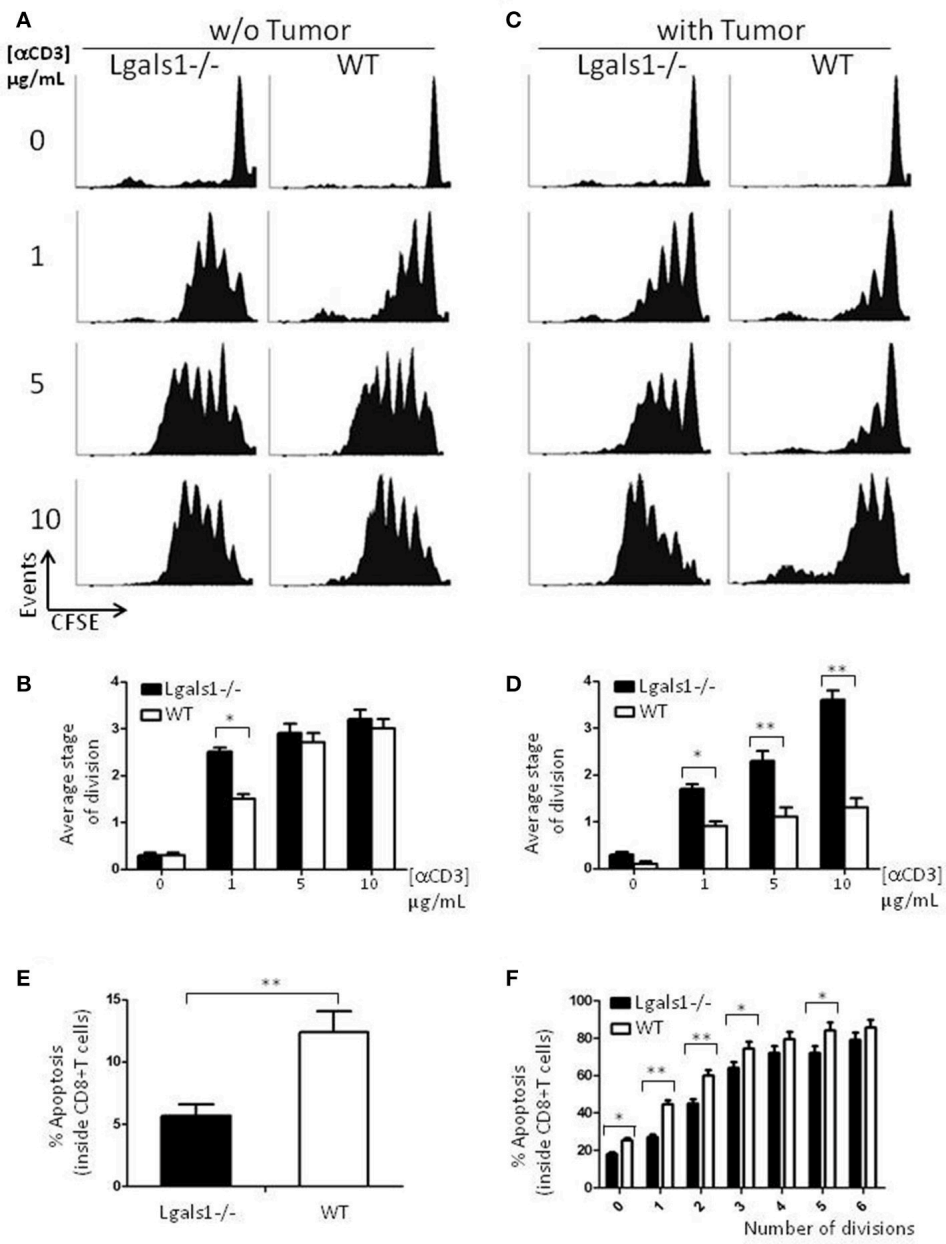
Role of endogenous Galectin-1 in the proliferation and activation-induced cell death of CD8+ T cells. Proliferation rates of wild-type and Lgals1-/- CD8+ T lymphocytes are compared upon activation with different anti-CD3 concentrations in a 72 h proliferation assay. **(A,B)** In the absence of TRAMP-C1 tumor cells. **(A)** Representative CFSE dilution proliferative experience. **(B)** Graph bars indicating the average stage of division obtained from three independent experiences; White bars: wild-type cells; Black bars: Lgals1-/- cells (*p < 0.05, *t*-test). **(C,D)** In the presence of 1% TRAMP-C1 tumor cells. **(C)** Representative CFSE dilution proliferative experience. **(D)** Graph bars indicating the average stage of division obtained from three independent experiences; White bars: wild-type cells; Black bars: Lgals1-/- cells (*p < 0.05, *t*-test). **(E)** Role of endogenous Galectin-1 in the apoptosis of CD8+ T cells upon polyclonal activation in the presence of 1% TRAMP-C1 cells. Apoptosis rates of wild-type (*n* = 3) and Lgals1-/- (*n* = 3) lymphocytes are comparatively analyzed by Annexin V staining inside total CD8+ T cell gate **(E)** or inside each peak of division (CFSE) in the CD8+ T cell gate **(F)**. White bars: wild-type cells; black bars: Lgals1-/- cells (*p < 0.05, *t*-test).

Besides tumor cells themselves, other cells in the tumor associated microenvironment play fundamental roles in the determination of the tumor evolution. Understanding how endogenous Gal-1 in each of these cells affects their own properties seems, therefore, essential to have a complete functional picture of the PCa microenvironment. In particular, little is known about the endogenous role of Gal-1 in immune cells. Therefore, we first checked whether Gal-1 is expressed in immune cells and compared its expression levels with those observed in tumor cells. Interestingly, the expression levels of Gal-1 in immune cells [T lymphocytes and antigen presenting cells (APC)] were considerably lower (10–20 times less) than those of tumor cells (Figures 1A,B). Low levels of Gal-1 expression in immune cells do not indicate *a priori* that lymphocyte endogenous Gal-1 does not play any role as an intrinsic modulator of their properties. Thus, we decided to design an *in vitro* experimental model where the function of Lgals1-/- lymphocytes would be compared with the wild-type (WT) ones in the presence or absence of wild type tumor (responsible of secreting high levels of “exogenous Gal-1”). As readout, we focused on the function of CD8+ T lymphocytes, as these cells are critical in the anti-tumor cytotoxic response (46). Our results demonstrate that without endogenous Gal-1, CD8+ T cells showed higher proliferative rates (Figure 2). A detailed analysis of these results shows that, in the absence of tumor, there was a trend toward greater proliferation of Gal-1-deficient CD8+ T lymphocytes mainly at low conditions of stimulation (Figures 2A,B, 1μg/mL anti-CD3). However, the results were considerably more significant in the presence of an immunosuppressive microenvironment generated by the tumor cells (Figures 2C,D); the absence of endogenous Gal-1 led to higher CD8+ T cell proliferative capacity even in such conditions. We highlight that, the major source of extracellular Gal-1 in these experimental settings are tumor cells that almost saturate the extracellular medium by the secretion of high quantities of Gal-1 [dosed by ELISA at levels of 20 ng/mL, supported also by the high cellular levels of Gal-1 shown in Figure 1, and evaluated in patients in our previous study (30) and reported at high levels in the serum of cancer patients (47–50)]. The most potent abrogation of tumor-immunosuppression was achieved by the absence of endogenous Gal-1 in lymphocytes combined with the down-regulation of Gal-1 in tumor cells (Figures 1C,D). While the immune effects of Gal-1 in tumor cells have been extensively evaluated (13–15, 51), the endogenous effect of Gal-1 effect in lymphocytes is original and requires more understanding.

Since a widely described action of exogenous Gal-1 is the induction of cell death on activated T lymphocytes (9), we evaluated if lymphocytes with no endogenous Gal-1 were differentially induced into apoptosis in our tumor experimental model. Figure 2E shows the percentage of apoptotic cells determined by Annexin V staining inside the CD8+ T cell subpopulation, and Figure 2F shows the same staining inside each peak of division of CD8+ T cells. Altogether, these results indicate that the absence of endogenous Gal-1 in CD8+T lymphocytes protects these cells from apoptosis in the presence of tumor cells. As in proliferative studies, the major source of extracellular Gal-1 was the tumor.

**Figure 3:**
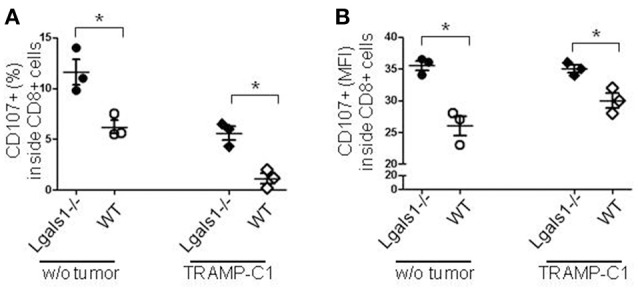
Role of endogenous Gal-1 in the degranulation capacity of CD8+ T lymphocytes. T lymphocytes from wild-type (empty circles and diamonds) or Lgals1-/- (filled circles and diamonds) mice were cultured in the presence of WT APC and 1% TRAMP-C1 tumor cells. Lymphocytes were stimulated by the CD3 receptor agonist antibody. After 48 h, the mobilization of CD107a was evaluated by flow cytometry. **(A)** Percentage of CD8+ T cells expressing CD107a. **(B)** CD107a mean intensity fluorescence (MFI) in CD8+ T cells (*p < 0.05, *t*-test).

Degranulation is a prerequisite to perforin-granzyme-mediated cytotoxic function by responding to antigen-specific CD8+T cells. The lytic granules are surrounded by a lipid bilayer containing numerous lysosomal-associated membrane proteins (LAMPs, CD107a and b); thus, labeling responding cells with anti-CD107 antibodies and measuring their expression by flow cytometry can directly identify degranulating CD8+ T cells. The results shown in Figure 3 demonstrate that the absence of endogenous Gal-1 in CD8+ T lymphocytes increases their ability to degranulate. This was also observed in conditions without tumor cells and at different percentages of TRAMP-C1 (Figure 3 and data not shown). Interestingly, endogenous Gal-1 in lymphocytes affects not only their percentages of degranulating cells (Figure 3A), but also the CD107a mean fluorescence intensity (MFI). This last result indicates that the intrinsic expression/exposition of CD107a is regulated by this lectin on a *per cell* basis (Figure 3B). Altogether, these results imply that endogenous Gal-1 in CD8+T cells an impotant role in the control of their function, particularly in the presence of tumor cells.

### Lymphocyte endogenous Gal-1 modulates their function independently of exogenous factors

**Figure 4:**
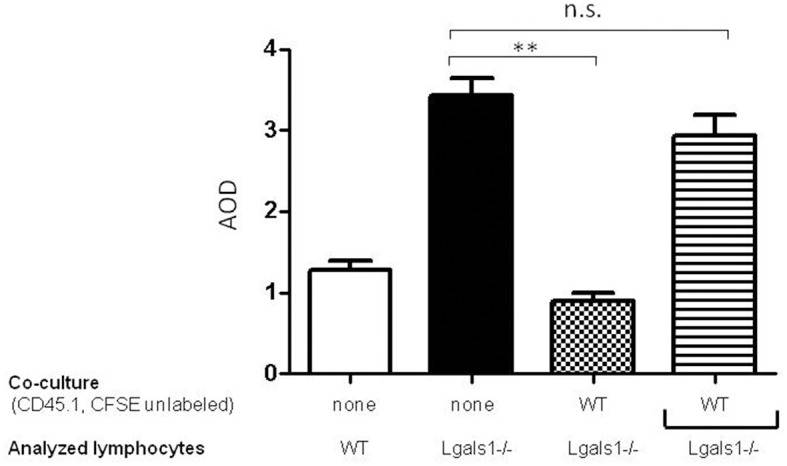
Endogenous Gal-1 regulates lymphocyte proliferation through cell-to-cell contacts. Lymphocytes from Lgals1-/- (black bars) and wild type (white bars) mice were polyclonally activated in presence of 1% TRAMP-C1 cells and 10% APC. Additionally, Lgals1-/- (CFSE labeled) and wild type (CD45.1; CFSE-) lymphocytes were co-cultured together or separated through a transwell. In all cases activation was performed by 0.25 μg/ml soluble anti-CD3 mAb. Proliferation was evaluated at 72 h by CFSE dilution in the CD8+ CD45.2+ gate. Results are expressed as average stage of division (AOD) and represent the mean + SD of three experiences. Statistic significance **p < 0.01 (Student *t*-test).

To evaluate if the immunosuppressive role of lymphocyte endogenous Gal-1 involves cell-to-cell contacts or the production of soluble factors, we performed transwell experiments. Figure 4 shows that Gal-1 deficient lymphocytes proliferate more extensively than wild-type ones when activated in the presence of tumor (as in Figure 2). We next co-cultured wild-type and Gal-1 deficient lymphocytes (they differed in the expression of CD45 isoform; both were activated and proliferation was evaluated in CD45.2+ CFSE labeled). The presence of wild-type cells reduced the proliferation of Gal-1 deficient CD8+T cells. However, when these two lymphocyte sources were separated through a transwell membrane, such a reduction in proliferation was no longer observed (Figure 4). This experiment clearly demonstrates the fundamental role of endogenous lymphocyte Gal-1 in controlling their function upon activation, a phenomena that is independent of the tumor secretion of Gal-1 and can be controlled through lymphocyte-lymphocyte interactions. This introduces new variants for the current understanding of the role of Gal-1 in defining a tumor-associated tolerogenic context.

### Gal-1 negatively regulates *in vivo* immunity against PCa

**Figure 5:**
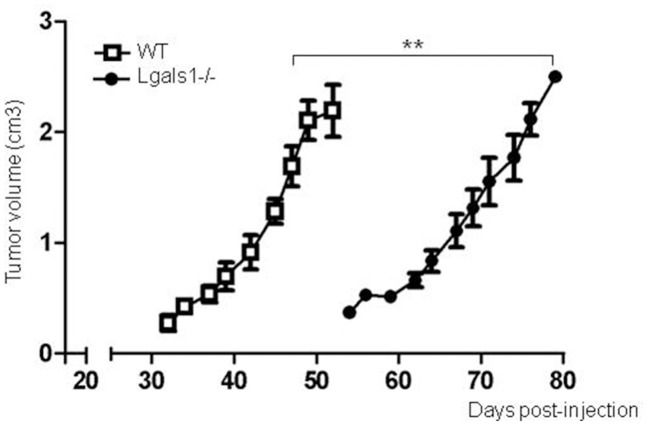
Endogenous Gal-1 in lymphocytes accelerates *in vivo* TRAMP-C1 tumor growth. Adult male nude mice were reconstituted with experienced immune cells from wild-type (white squares; *n* = 5) and Lgals1-/- (black circles; *n* = 6) mice. The day after, the mice received 1 × 10^6^ TRAMP-C1 in Matrigel®, and tumor growth was monitored by volume measures. **p < 0.01, *t*-test.

Previously, it has been reported that several tumor cells lose their tumorigenicity when injected into Lgals1-/- mice, compared with their injection into wild-type mice (28, 29). We obtained the same behavior in the case of the TRAMP-C1 prostate tumor model. In fact, while 92% of wild-type mice injected *s.c*. with TRAMP-C1 cells developed tumors (lag time: 47 ± 5; duplication time: 7 ± 2 days, *n* = 5), no Lgals1-/- mice (0/4) developed tumors after up to 80 days. However, the conclusions obtained from this type of experimental model are complex, and the real impact of immune-mediated effects cannot be established since Gal-1 is absent in all cells of the body, including endothelial cells in which it was previously demonstrated Gal-1 plays a major regulatory role. To evaluate more precisely whether endogenous Gal-1 in lymphocytes affects the capacity of mice to control tumor growth under *in vivo* settings, we developed the following experimental procedure: anti-tumor lymphocytes were induced by the immunization of Lgals1-/- and wild-type mice with dendritic cells pulsed with TRAMP-C1 lysates. At day 7 post-immunization, immune cells were obtained from the BAIM lymph nodes; no significant phenotype changes were detected at this stage (total absolute cell numbers, %CD4+, %CD8+, %CD4+CD25+Foxp3+Tregs; data not shown). Lymphocytes obtained from the lymph nodes of immunized mice (wild type or Lgals1-/-) were then transferred into an immunodeficient nude mouse (1-to-1 mice ratio). Under such reconstitution settings, all non-immune host compartments (e.g., stroma and particularly endothelial cells) expressed Gal-1 at normal levels, with the exception of T lymphocyte (transferred) cells. This system allowed us to compare the ability of Lgals1-/- and wild-type lymphocytes to control tumor growth in a WT micro- and macro-environment. An in-depth comparison analysis demonstrated that mice reconstituted with Lgals1-/- lymphocytes showed a marked increase in tumor latency and a slight decrease in tumor duplication time (Figure 5 and Table 1). In contrast, tumorigenesis, defined as the percentage of mice developing tumors, was 100% in both experimental groups, implying that endogenous Gal-1 on lymphocytes plays an important role in controlling tumor growth in initial phases, but later other molecular pathways can circumvent its action.

**Table 1:** Different Tumor Growth on Nude Mice Reconstituted with Gal-1-deficient or competent lymphocytes.

	**Latency (days)**	**Duplication time (days)**
WT lymphocytes	32 ± 2	6.0 ± 1.2
Lgals1-/- lymphocytes	54 ± 4	8.2 ± 0.4

### Cellular mechanisms involved in lymphocyte modulation by endogenous Gal-1

**Figure 6:**
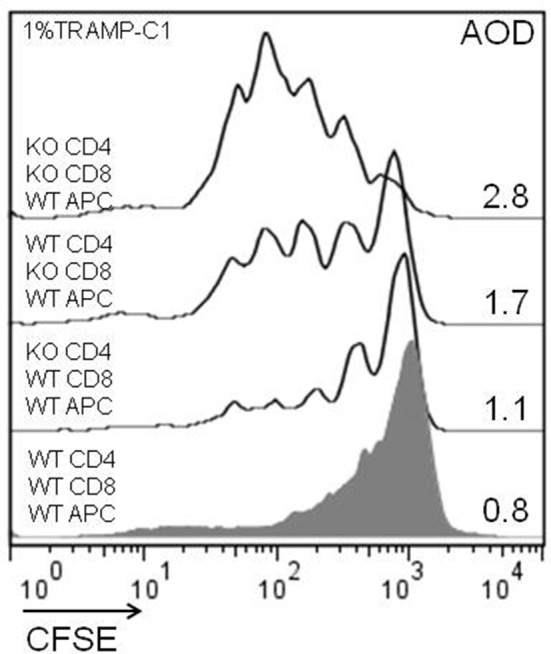
Cellular mechanisms involved in the antitumor effect of lymphoid endogenous Gal-1. Wild-type and Lgals1-/- CD4+, CD8+ (cell sorted, purity >98%), and APC (adherence, purity CD14+ >90%) were combined and co-cultured with 1% TRAMP-C1 tumor cells. The lymphocytes were activated with anti-CD3mAb. The proliferative rates of CD8+ T cells in different cellular combinations were evaluated by CFSE-dilution in a 72-h essay. AOD: average of division. One experiment out of three with similar results is shown.

Considering the previous *in-vitro* and *in-vivo* results, we analyzed the impact of the endogenous Gal-1 absence in the different subpopulations of cells involved in the immune response. For this purpose, we purified APC, CD4+, and CD8+ T cells to homogeneity and performed *in-vitro* proliferation assays by mixing the subpopulations of different genotypes in fisiological proportions. The results shown in Figure 6 demonstrate that the absence of Gal-1 in CD4+ and CD8+ T cells results in higher proliferative rates of CD8+ T lymphocytes. Higher effect was observed when Gal-1 was absent in the responding CD8+ T cells compared to CD4+ T cells or all wild-type immune cells (AOD = 1.7 vs. 1.1 or 0.8, respectively); however, maximal CD8+ T cell proliferation occurs when Gal-1 is absent in both lymphoid subpopulations (AOD = 2.8). The absence of Gal-1 in APC did not produce any change in the proliferative potential of CD8+ T lymphocytes (data not shown). Altogether, these results clearly demonstrate that endogenous Gal-1 regulates the functional properties of CD4+ and CD8+ T cells.

## Discussion

The pleiotropic functions of Gal-1 are partly due to the complex cellular distribution of this lectin. At the extracellular level, Gal-1 is able to interact with glycosylated receptors (through their N-acetyllactosamine glycans), thereby forming complex membrane lattices [reviewed in (52)]. Through this outside-in process, Gal-1 regulates several cellular properties, acting as a negative regulator of immune responses [reviewed in (53, 54)]. Consistently, most of the current Gal-1-based immune strategies proposed for autoimmunity and cancer are focused at the extracellular level.

Additionally, the intracellular regulation of Gal-1 is expected to perform extra regulatory functions. At this subcellular localization, Gal-1 regulates several cellular processes, such as growth, survival, and differentiation; several of these effects are carbohydrate-independent [reviewed in (52)]. It has been demonstrated that the downregulation of the endogenous Gal-1 expressed by tumor cells has a direct effect on the immune response [our results and (13–15, 51)]. As expected, these strategies also modify the extracellular levels of Gal-1 (38), as the transformed cells are the main Gal-1 producers. Interestingly, Gal-1 is also expressed by other cells of the tumor microenvironment; the endogenous function of Gal-1 in those cells may affect tumor growth. Indeed, several reports have demonstrated the decreased tumorigenic potential of various tumor cell lines when injected into Gal-1-deficient mice (28, 29). The absence of Gal-1 on endothelial cells of Lgals1-/- hosts is responsible for the loss of tumorigenicity; angiogenesis defaults prevent an adequate supply of nutriments, oxygen, and hormones, factors that are essential for optimal tumor growth (28). While this report does not exclude the importance of this scenario, it proposes a complementary alternative in which tumors require Gal-1 to be endogenously expressed in T lymphocytes to escape from immune attack.

In fact, our results demonstrate that lymphocytes isolated from Gal-1-deficient mice have higher CD8+ T cell proliferative rates and effector functions, even when activated in the presence of high levels of extracellular Gal-1 secreted by tumor cells. This biological observation could be due to a direct endogenous effect of Gal-1 on CD8+ T cells or through indirect effects on other cell types; this report dissected the functional role of endogenous Gal-1 in immune cells. Our results show that the expression of Gal-1 on antigen-presenting cells is indifferent at potentiating CD8+ T cell functions. However, the absence of Gal-1 in CD4+T cells induced higher levels of CD8+ T proliferation. Gal-1 plays a fundamental role in the control of differentiation and function of conventional CD4+CD25+Foxp3+ Tregs (17). While we did not detect changes in the frequency of Tregs in our experimental settings (data not shown), such Tregs are functionally deficient (17). Consequently, CD4+T cell subpopulations from Lgals1-/- and wild-type mice differ at least at this functional level. However, other endogenous modifications are also plausible in the CD4+T cell compartment, as was previously demonstrated in parasitic models of infection (21, 22). More interestingly, the absence of endogenous Gal-1 induces higher functional properties of CD8+ T cells. These results agree with a previous report that demonstrated that Gal-1 has a major impact over proliferation burst, lymphocyte survival upon activation, and effector fate (23).

Mechanistically, our transwell experiments demonstrated that the immunoregulation mediated by endogenous Gal-1 in immune cells involves cell-to-cell interactions. In fact, the co-culture of Gal-1 deficient together with wild-type lymphocytes abolished the immunopotentiation observed in Lgals1-/- cells alone. In addition, our results demonstrate that soluble factors are not involved in this type of regulation. Altogether, these results led us to hypothesize that Gal-1 deficient lymphocytes lack immune-inhibitory membrane receptors (or, alternatively, acquire immune-activating receptors) that have a direct effect on lymphocyte function. It has been reported that intracellular Gal-1 could affect cell behavior through regulation of the transcriptional machinery (Gal-1 forms part of the spliceosome), miRNA, and signaling pathways (52). Effects coming from intracellular Gal-1 interactors in lymphocytes have not yet been investigated.

Interestingly, the relevant roles of lymphocyte endogenous Gal-1 have recently been proposed in active systemic lupus erythematosus. In fact, patient lymphocytes demonstrate lower levels of endogenous Gal-1 upon activation, a mechanism that may make them prone to autoimmunity (55). In cancer, our results support a scenario where the high levels of exogenous Gal-1 produced by tumor cells are not enough to evade immune attack; tumors require additional licensing by lymphocyte endogenous Gal-1 to be efficiently immune-suppressive. These results define a new and potent target for immunotherapy in PCa. From a clinical point of view, it is interesting to note that the immunopotentiation resulting from the downregulation of Gal-1 in lymphocytes is more significant in the presence of a tumor; consequently, an anti-PCa therapeutic strategy based on these results should have few side effects.

## Author contributions

EC and GC performed and analyzed the work. DC data interpretation, critical revision. DL conceived the idea, analyzed data, supervised the project and wrote the paper. All authors discussed the results and contributed to the final manuscript.

### Conflict of interest statement

The authors declare that the research was conducted in the absence of any commercial or financial relationships that could be construed as a potential conflict of interest.

## Abbreviations

PCa: prostate cancer
Gal-1: Galectin-1
TRAMP-C1: prostate tumor cell line used in this preclinical model
WT: wild type mice
Gal-1 KO: (Lgals1-/-) Galectin-1 deficient mice
AOD: average stage of division
CFSE: Carboxyfluorescein succinimidyl ester used to trace cell division by flow cytometry
Th: T helper profile of the immune response
DC: dendritic cells
Treg: regulatory T cells
SDS-PAGE: sodium dodecyl sulfate-polyacrylamide gel electrophoresis
BAIM: brachial, axillary inguinal and mesenteric lymph nodes.
